# The (Mis)Information Game: A social media simulator

**DOI:** 10.3758/s13428-023-02153-x

**Published:** 2023-07-11

**Authors:** Lucy H. Butler, Padraig Lamont, Dean Law Yim Wan, Toby Prike, Mehwish Nasim, Bradley Walker, Nicolas Fay, Ullrich K. H. Ecker

**Affiliations:** 1 School of Psychological Science, University of Western Australia, Crawley, WA Australia; 2 School of Engineering, University of Western Australia, Crawley, WA Australia; 3 School of Physics, Mathematics and Computing, University of Western Australia, Crawley, WA Australia; 4 Public Policy Institute, University of Western Australia, Crawley, WA Australia

**Keywords:** Experimental control software, Misinformation, Social media

## Abstract

Given the potential negative impact reliance on misinformation can have, substantial effort has gone into understanding the factors that influence misinformation belief and propagation. However, despite the rise of social media often being cited as a fundamental driver of misinformation exposure and false beliefs, how people process misinformation on social media platforms has been under-investigated. This is partially due to a lack of adaptable and ecologically valid social media testing paradigms, resulting in an over-reliance on survey software and questionnaire-based measures. To provide researchers with a flexible tool to investigate the processing and sharing of misinformation on social media, this paper presents The Misinformation Game—an easily adaptable, open-source online testing platform that simulates key characteristics of social media. Researchers can customize posts (e.g., headlines, images), source information (e.g., handles, avatars, credibility), and engagement information (e.g., a post’s number of likes and dislikes). The platform allows a range of response options for participants (like, share, dislike, flag) and supports comments. The simulator can also present posts on individual pages or in a scrollable feed, and can provide customized dynamic feedback to participants via changes to their follower count and credibility score, based on how they interact with each post. Notably, no specific programming skills are required to create studies using the simulator. Here, we outline the key features of the simulator and provide a non-technical guide for use by researchers. We also present results from two validation studies. All the source code and instructions are freely available online at https://misinfogame.com.

The rise of the Internet and social media has changed the way people consume information (Flanagin, 2017). To illustrate, according to a survey conducted by Pew Research Center (2021), approximately half of U.S. residents frequently use social media platforms as a primary news source. Moreover, although people can intentionally seek out information on social media, people are often exposed to information incidentally, due to it being algorithmically curated, or shared or interacted with by others in their social network (Fletcher & Nielsen, 2018; Nikolov et al., 2019). The social media information environment is characterized by a dependency on social connections (Metzger et al., 2010): On social media, anyone can produce and share information (Ciampaglia et al., 2015; Flanagin, 2017), and information flow often involves many small-scale, bidirectional information transfers. This contrasts with traditional media in which information flow tends to be unidirectional, from institution to a large number of consumers (i.e., one-to-many; Flanagin, 2017).

Although social media has a number of benefits—for example, it has facilitated the uncensored spread of vital information to previously inaccessible portions of the population (e.g., during natural disasters; Finch et al., 2016)—it also allows for the easy production and dissemination of misinformation^1^ (e.g., Allcott et al., 2019; Wang et al., 2019). Given the negative individual and societal implications belief in misinformation can have (e.g., exacerbating vaccine refusal and climate-change denial; Donzelli et al., 2018; Loomba et al., 2021; Simonov et al., 2022; van der Linden, 2015), substantial research has gone into understanding the conditions under which people form misinformed beliefs, and reject or accept corrective information (for reviews, see Ecker et al., 2022; Pennycook & Rand, 2021; van der Linden, 2022). However, despite the shift in real-world information consumption, many of the paradigms used to study informational influence look much the same as they did decades ago (Walter & Tukachinsky, 2020).

Specifically, experimental research on misinformation typically follows the same basic paradigm: Participants are presented with a piece of misinformation (often presented as a news headline or in the context of a [fictional] news report) and are required to rate their level of belief in said misinformation on a Likert scale. These studies typically also involve presenting participants with an intervention, either prior to (i.e., pre-bunking) or following (i.e., debunking) misinformation exposure. Although such research allows for inferences about the cognitive processing of misinformation, as well as ways to reduce misinformation belief, these studies tend to view the participant as a passive information consumer, rather than an active consumer and producer of content, thereby overlooking how social and cognitive factors influence misinformation-propagation behaviors (Flanagin, 2017; Weeks & Gil de Zúñiga, 2021). Further, the misinformation (and, if present, corrections) is typically presented in isolation from social information (e.g., information about the source, or engagement metrics such as user comments or “likes”), thus systematically discounting how social factors can influence information processing. Given the high reliance on social media platforms for information consumption, and the potential negative ramifications of misinformation propagation, understanding these factors is necessary to counteract the threat of misinformation.

Studies that have attempted to empirically assess misinformation propagation and/or engagement behavior typically use relatively rudimentary measures, such as questionnaires similar to those used in the belief-focused research (e.g., X. Chen, 2016; Globig et al., 2022; MacFarlane et al., 2021; Pennycook et al., 2021). Specifically, in these studies, participants are usually presented with a static post (e.g., a claim or a headline) and are required to rate their intent to engage with it or share it. This intent is measured in one of two ways: Firstly, intent can be measured as an expression of sharing likelihood, typically on a Likert scale ranging from “Very unlikely [to share]” to “Very likely [to share]” (e.g., Mena, 2020), or using a limited scale asking participants if they would *consider* sharing the post (e.g., a three-point scale with response options “no”, “maybe”, and “yes”; e.g., Pennycook et al., 2020; Pennycook & Rand, 2019). This approach provides a crude measure of sharing behavior (that is, one would assume a positive correlation between a person’s self-reported likelihood of sharing and their actual sharing behavior)^2^ and is arguably useful to gauge factors such as people’s belief certainty or their perception of others’ endorsement of a claim. However, making inferences from these data to an individual’s actual misinformation-propagation behavior is inherently flawed. Not only are such measures likely confounded by social-desirability bias, but responses to such scales do not directly map on to actual sharing behavior given sharing is a binary outcome (Mosleh et al., 2020). Additionally, such measures do not provide information about other forms of engagement behavior (especially engagement behaviors that signal “dis-endorsement”, such as flagging or fact-checking) which may occur when people encounter misinformation (or other types of information) online.

A second approach to measuring misinformation propagation is the use of a forced-choice misinformation-propagation measure (e.g., MacFarlane et al., 2021). Specifically, participants are presented with a screenshot of a (mock) social media post and asked to indicate how they would engage with the post from a series of options (e.g., “share”, “like”, “flag”, or “pass”). Although this more closely approximates actual online engagement behavior, such measures have low face validity. Additionally, their purely hypothetical nature means that they lack the potential social consequences present in the real world (e.g., reputation damage arising from endorsing false information; Altay et al., 2022), which may impact how people engage with content online (MacFarlane et al., 2021; Mosleh et al., 2020).

Another strand of research has used gamified tools that have been developed primarily for the purpose of educating users about common misinformation characteristics or misinformant tactics. Specifically, games such as Fake It To Make It (Urban et al., 2018), Bad News (Roozenbeek & van der Linden, 2019), Harmony Square (Roozenbeek & van der Linden, 2020), Go Viral! (Basol et al., 2021), and Cranky Uncle (Cook et al., 2022) teach players common techniques for promoting misinformation in the hope that this has an inoculating effect (van der Linden et al., 2020). They generally also allow for data collection and can thus be used for research purposes, for example to test if players develop misinformation resilience. Similarly, the Fakey platform (Micallef et al., 2021) creates a simulated interactive timeline by scraping the web for high- and low-quality information with the aim of increasing media literacy by providing participants with feedback based on how they interact with each type of information. However, while these tools provide insights into information processing in a context more closely resembling the real world, they are not freely adaptable, and this lack of flexibility limits their utility as research tools.^3^

To overcome the constraints and limitations of past research and paradigms, we have developed The Misinformation Game: a dynamic social media simulator that mimics key characteristics of well-known platforms while also allowing for direct and flexible control and manipulation of key variables (see Fig. 1). Specifically, the social media simulator allows researchers to present participants with mock social media posts in a relatively natural setting. Researchers have the capacity to manipulate (1) the posts and how they are presented (i.e., as text, static image, animated gif, or a combination, as well as whether posts are presented on separate pages or in a feed), (2) source information (e.g., a source’s name, avatar, follower count, and a credibility score), and (3) engagement information (e.g., number of times a post has been liked, disliked, shared, flagged as misleading or otherwise inappropriate, or commented on). Additionally, participants can engage with posts in ecologically valid ways by choosing an engagement behavior (with options again including liking, disliking, sharing, flagging, and commenting), and can receive dynamic feedback (i.e., changes to their own simulated follower count and credibility score) depending on how they interact with both true and false posts. This allows researchers to: (1) assess how social-context information provided on social media platforms can influence people’s beliefs and behaviors, (2) study misinformation-propagation (or suppression) behaviors in a realistic setting, and (3) assess how social feedback can influence subsequent belief and engagement behaviors. Example games (one presents posts on separate pages, the other presents posts in feed mode) are available from https://misinfogame.com/link/ExampleGame.^4^

**Fig. 1:**
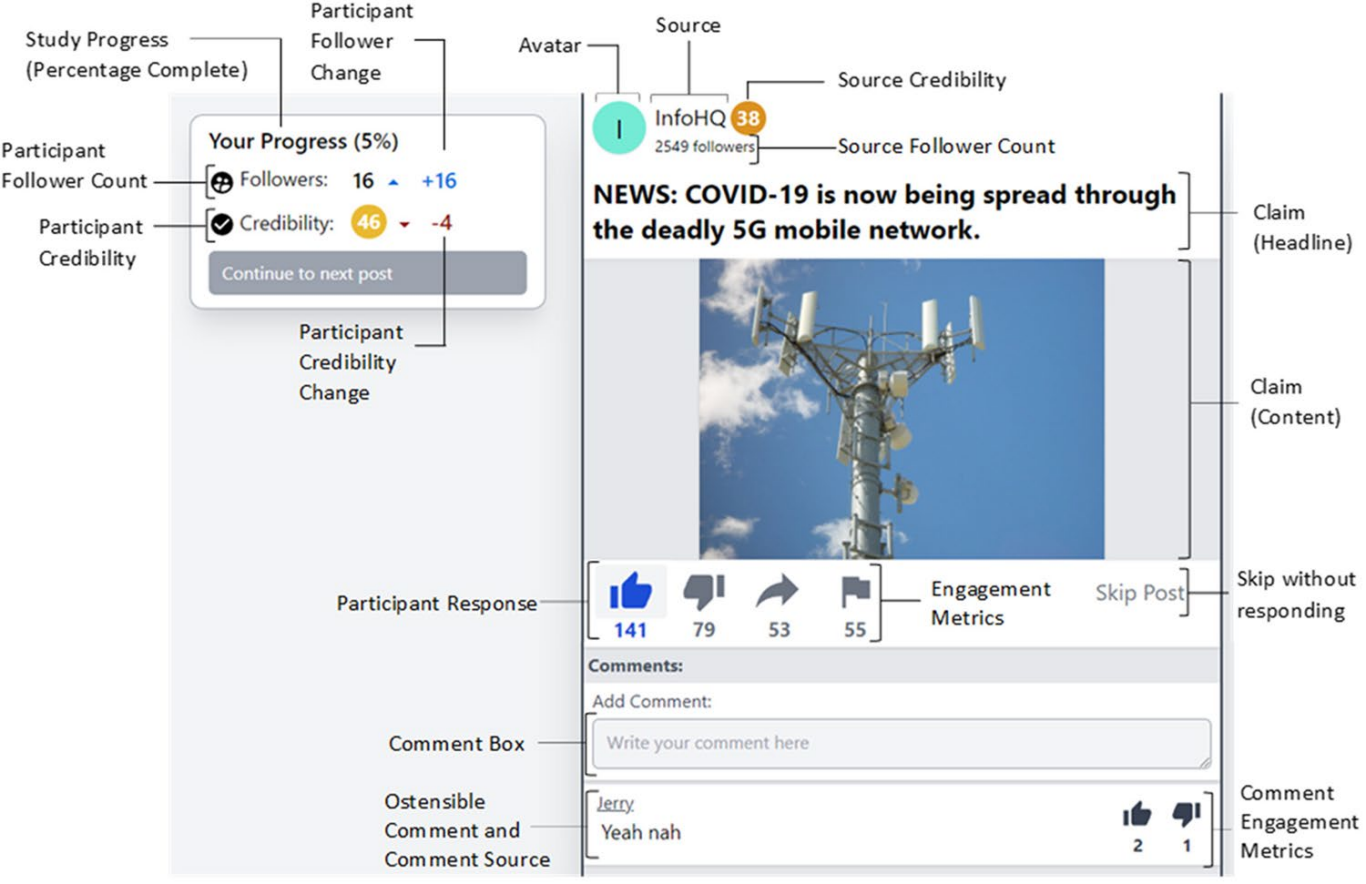
Example of a (false) post presented in the Misinformation Game. The example is from a simulation with all features enabled. Engagement metrics, follower counts and credibility scores, post headline (claim) and content (photo), and avatar/source information can be altered or toggled on and off using a Google Sheets template (see text for further details)

Importantly, the platform has been created such that the key variables of interest can be easily manipulated or disabled using a Google Sheets spreadsheet template (accessible from https://misinfogame.com/link/StudyTemplate), and as such the tool can be used by researchers with little or no coding experience. In the following section, we provide an in-depth overview of the key characteristics of social media platforms, and their potential influence on information-veracity judgments, as well as how the Misinformation Game can be used to assess how these factors influence misinformation belief and sharing. Next, we provide instructions for setting up a study using the simulator, as well as how to integrate these studies into the online survey platform Qualtrics. We then outline two studies run using the social media simulator: The first study provides a validation of the simulator as a research tool, and the second study provides information regarding participant perceptions of the simulator.

## Key characteristics of social media and the Misinformation Game

### Source information

When information sources are known and familiar, such as established news institutions or friends and family, information consumers can easily assess source credibility. However, information sources in the social media environment may be unknown, unfamiliar, or anonymous, and therefore it can be difficult for information consumers to accurately assess source credibility (Metzger et al., 2010; Metzger & Flanagin, 2013). In situations where traditional cues of source credibility are unavailable, people may turn to other source-related information to gauge information quality (e.g., a source’s follower count or prestige; Mena et al., 2020; Metzger & Flanagin, 2013). Past misinformation research has provided evidence for the role of source credibility when evaluating information veracity (e.g., Ecker & Antonio, 2021; Kim et al., 2019; Nadarevic et al., 2020; Walter & Tukachinsky, 2020; but also see Dias et al., 2020; Wintersieck et al., 2021). However, exactly how people make these judgments on social media—and how they assess the value of information from different sources—is unclear.

The Misinformation Game allows researchers to directly manipulate source information, including account names, avatars, and follower counts to directly assess these factors. In addition, it allows for the inclusion of an explicit credibility badge that provides a score from 0 to 100. Although not currently present on real-world social media platforms, similar ratings are seen in e-commerce (e.g., eBay, Uber) and have been shown to influence consumer behavior, such as willingness to buy from a seller (Flanagin et al., 2011). Moreover, the social media simulator lets researchers flexibly control how posts are paired with sources, from entirely random to fully specified, with intermediate options to probabilistically allocate posts to sources. This can enable research on the impact of source-related information on information believability (e.g., comparing sources with high vs. low follower counts, different avatar characteristics, etc.), or on the utility of source credibility metrics for combatting misinformation spread (Prike et al., 2023).

### Engagement information

Another aspect of social media platforms that may influence credibility and veracity judgments is the presence of engagement metrics. Engaging with news posts can be used to signal support of (likes, shares) or opposition to (dislikes, flags) the information presented, and thus engagement metrics may be used by information consumers as a proxy for social endorsement (i.e., the bandwagon heuristic; Mena et al., 2020; Metzger & Flanagin, 2013; Peters et al., 2013). As such, people may be more vulnerable to misinformation that is highly endorsed (Borah & Xiao, 2018; Butler et al., 2022; Vlasceanu & Coman, 2021). In fact, it has been argued that, if perceived as normative, endorsement may drive people to engage with information they do not believe, due to fear of social exclusion or the belief that engaging will lead to social inclusion (Brown et al., 2022; Ren et al., 2023; Van Bavel et al., 2021).

The Misinformation Game allows researchers to flexibly alter the number of likes, shares, dislikes, and flags associated with any given post, as well as the display of a customizable set of comments (which themselves can be associated with a number of likes and dislikes). Engagement options can be individually toggled on and off, allowing researchers to isolate the influence of specific engagement metrics (for example, the dislikes, shares, and flags can all be disabled to allow researchers to assess how likes alone influence belief and sharing). This enables research into whether, and under what conditions, endorsement may drive the sharing of information, and also the relative influence of different engagement metrics. For example, the social media simulator could be used to assess whether a negativity bias exists when processing misinformation on social media, such that negative engagement metrics (e.g., number of dislikes) are more influential than positive engagement metrics (e.g., number of likes; Bebbington et al., 2018). Quantity of engagement (i.e., the displayed number of likes, etc., on a post) can also be disabled in studies where this information is not required.

### Behavior choices and dynamic social feedback

Social media enables one-to-one and one-to-many social interactions. Users can signal to others their endorsement (or dis-endorsement) of information, thereby contributing information to the news environment. These behavior choices do not necessarily map onto beliefs in a straightforward manner (Metaxas et al., 2015). That is, although belief changes are often assumed to be directly associated with changes in sharing and engagement behaviors, there may be other motives that prompt this behavior (e.g., to signal group membership or support of a political leader; Van Bavel et al., 2021). Rather than relying solely on measures of belief change, research has started to examine misinformation transmission and fact-checking behaviors; however, as stated previously this often involves relatively unrealistic methods (e.g., MacFarlane et al., 2021, presented screenshots of mock social media posts and asked participants to indicate their preferred behavior choice via a multiple-choice item). While these methods may approximate behavior choices on social media, the ecological validity of the measures is questionable (Mosleh et al., 2020).

One key factor that previous methods typically disregard is that behavior choices made online can have consequences. For example, social media users can receive social feedback (negative, positive, or neutral) based on how they engage with or promote information (Van Bavel et al., 2021; also see Ren et al., 2023). This feedback can be direct (e.g., someone liking your comment) or indirect (e.g., loss of followers after liking a misleading post). Although it is well established that beliefs and behaviors can be influenced by the social environment (e.g., Fay et al., 2021; Smith & Semin, 2007), the impact of social feedback on information appraisal and propagation on social media is often overlooked (Weeks & Gil de Zúñiga, 2021). Further, studies investigating how social feedback influences engagement behavior typically use some form of financial incentive as part of the manipulation (e.g., a monetary “prize” for obtaining the most likes; Ren et al., 2023), making it difficult to isolate the effect of social factors.

The Misinformation Game allows researchers to define a set of behavioral response options that map onto the engagement metrics, in line with real-world platforms such as Twitter (e.g., if the researcher chooses to only offer “like” and “share” response options, the simulation will provide engagement metrics for only those two options, showing how often each post has been liked and shared). The platform can also provide participants with individualized, dynamic feedback (updated after each post is either submitted or scrolled off the page) based on how they interact with the presented posts. Researchers can control how participants’ follower counts and credibility scores change in response to their engagement with posts (e.g., liking a false post could increase a participant’s follower count and decrease their credibility score). This feedback can be specified separately for true and false posts, allowing researchers to create conditions where misinformation is favored or disfavored to differing degrees. Exact changes to participants’ follower count and credibility score can also be specified for each post to allow for more control. Thus, researchers can use the social media simulator to examine how people decide to interact with posts, based on belief and accuracy motives on the one hand (e.g., sharing only posts thought to be true), and social motives on the other (e.g., sharing to maximize follower count), as well as how these decisions and behaviors may change in response to the (dis)incentive structure of the system (i.e., how people are rewarded or punished for their engagement behavior). We note that these features can be disabled, and the tool can simply be used to display posts to participants in an engaging and ecologically valid manner.

## Study configuration: A step-by-step tutorial

Given that researchers who study misinformation come from a diverse range of disciplines (e.g., psychology, communication, political science, mathematics), we wanted to ensure the platform could be adapted and customized to fit a wide range of research needs—in fact, the platform may also be useful for research outside of the misinformation field. We also wanted to ensure the platform can be easily used by all researchers irrespective of their programming skills. Thus, all basic modifications to the study design can be achieved without programming skills, using a simple Google Sheets template. The platform is freely available at https://misinfogame.com. The information available includes all instructions and documentation, as well as the platform code, such that researchers with programming skills can adapt the platform further, as required. The following section provides a non-technical overview and a brief guide for installing and configuring the backend of the social media simulator, appropriate for researchers with no coding experience. For more in-depth information, documentation outlining all the functions of the Misinformation Game is available at https://misinfogame.com. Additionally, researchers have the capacity to leave comments or ask questions directly on the GitHub repository or the Slack channel.

### Installation

Prior to running studies using the social media simulator, researchers will need to install a number of applications and software (specifically: Visual Studio Code, Node.js and NPM Install, as well as the code base from GitHub) and set up a Google Firebase account for hosting their instance of the Misinformation Game website and securely storing its data. The installation process should take no more than an hour or two, and researchers who do not wish to edit the platform code will not need to go back to the installation process at any point. A step-by-step non-technical installation guide is available at https://misinfogame.com/NonTechnicalInstallation; for those familiar with command line and installing technical tools, a more concise technical guide is available at https://misinfogame.com/TechnicalInstallation. During the set-up process on Firebase, researchers are required to create a name for their application. This name will be used as the default base URL for all studies run from the application, and as such will be visible to participants.^5^ It is important to note that the backend installation only needs to occur on one computer: once the instance of the Misinformation Game has been set up on one computer the backend can be accessed online: multiple users (e.g., members of a lab) can be added as administrators, giving them full access to a secure backend administration dashboard to upload and enable studies on any computer.

### Configuring the study design

All studies using the default setup can be configured using the Google Sheets template that can be copied from https://misinfogame.com/link/StudyTemplate. The template consists of eight worksheets, six of which can be customized by the researcher to fit their specific research needs. Note that all cells that can be edited have a ‘help’ cell which describes their unique function in more detail. We provide an overview of the functions below.

#### About

The About sheet is not configurable by the researcher, and simply provides a general overview of how to use the template, including information about what each sheet in the template does, as well as a legend of cell types. This sheet should always remain unchanged.

#### Overview

The Overview sheet is not configurable by the researcher. It provides an overview of the researcher’s specific study design based on their input in other sheets within the template. This sheet also provides a study ‘status’, which indicates whether there is an error in the current setup. The information in this sheet will update based on changes made in the six editable sheets.

#### General

The General sheet provides information about the overall configuration of the study and is the first sheet that requires researcher input. The sheet is divided into three sections. (1) The Basic settings require researchers to enter general information about the study (e.g., the study name, the number of posts displayed to participants, whether or not reactions or comments are required by participants, and whether participants can select multiple reaction options for a single post). We note that if a study does not require participants to interact with posts, researchers can set both “Require Reactions” and “Require Comments” to ‘No’ under the basic settings, thus allowing participants to continue past simulated posts without engaging with (or explicitly selecting to skip) them.

(2) The User Interface settings allow the researcher to enable or disable platform features displayed to participants. For example, researchers can enable or disable whether participants can like, share, dislike, or flag posts, as well as whether follower and credibility information are displayed to the participant. Each interface option can be toggled on and off separately by entering ‘Yes’ or ‘No’ into the relevant cell. This is also where researchers can set whether posts are displayed in a feed (as they would be on typical social media platforms) or one-by-one (to allow for greater experimental control).^6^ Depending on the display mode, posts are either submitted by scrolling down the page until the post leaves the screen (in case of the feed display), or by the participant clicking “Continue to next post” (in case of the single-post-per-page display). Post submission triggers the relevant changes to follower count and credibility score (if those features have been enabled).

Finally, (3) the Advanced settings allow researchers to fine-tune aspects of how the study runs. For example, researchers can set a minimum time a post must be displayed for before a participant can advance to the next post (in the single-post display), and specify whether a completion code should be generated for participants.

#### Pages

The Pages sheet allows researchers to input the instructions unique to their specific study. There are four separate pages that can be edited: (1) the instructions displayed prior to the game rules, (2) the game rules themselves, (3) the instructions displayed after the game rules, and (4) the debrief sheet. Text can be edited (e.g., color, bold, italics, underlined) and each page can be disabled by removing all text in the relevant cell. Relevant game icons can be included in the game-rules page by using the placeholders defined in the cell to the right of the page content cell (e.g., to include the “like” thumbs-up symbol, researchers can input {{LIKE}}). A completion code can be included at any position on the debrief page by using either {{COMPLETION-CODE}} or {{COMPLETION-CODE-WITH-CONFIRMATION}} (the latter option will require participants to confirm they have read the associated debriefing before receiving their completion code). If necessary, images or hyperlinks can be included in each of the pages using HTML.

#### Sources

**Fig. 2:**
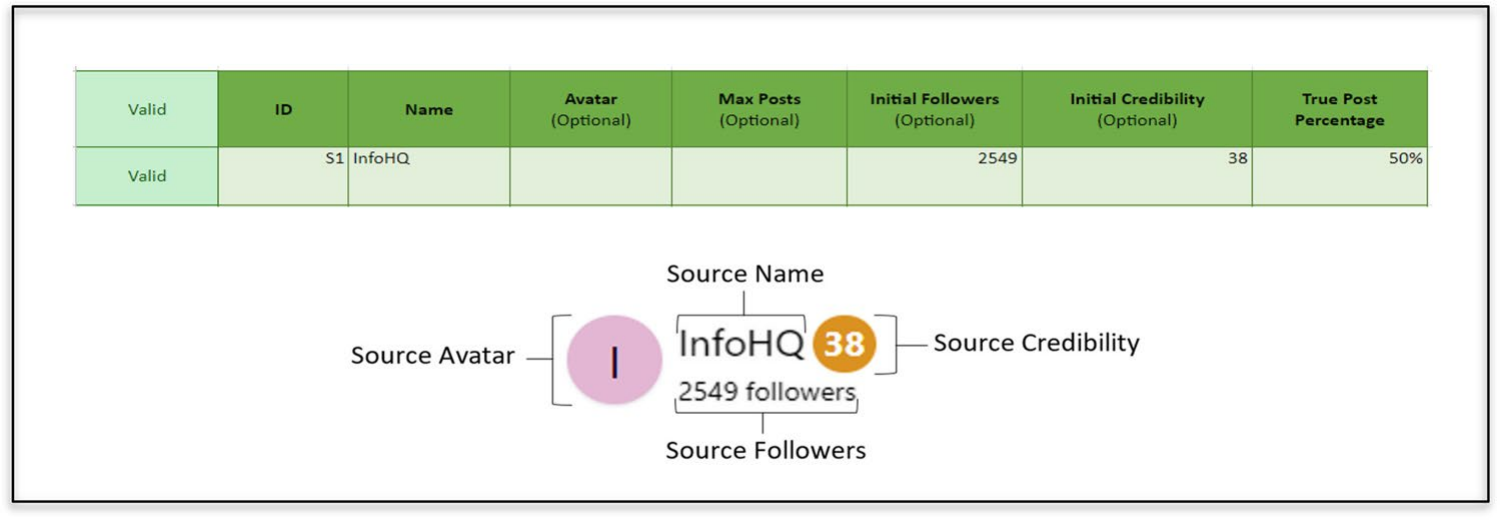
Example backend source configuration (*top*) and corresponding participant source view (*bottom*)

The Sources sheet allows researchers to set the information for each source used in the study (see top section of Fig. 2), specifically the source ID, name, avatar, maximum number of posts to be presented from this source, initial follower count and credibility score. The source ID is for backend use by researchers and allows for sources to be paired with specific posts; this ID is never displayed to participants. As such, researchers should feel free to use descriptive source IDs; for example, if sources are set to vary on initial credibility, using IDs to reflect this difference may be beneficial (e.g., “highcred1”, “lowcred1”). The lower panel of Fig. 2 shows the associated participant view. It is important to note that as no avatar was specified, the default avatar (first letter of name in a randomly colored circle) is displayed. If researchers wish to specify an avatar, this can be achieved by inserting an image into the cell (via Insert → Image → Insert an image in the cell).

If researchers do not wish to specify the initial follower number and credibility score for each source, the corresponding cells can be left blank; values will then be randomly sampled from normal (or skew-normal) distributions defined by the “default source values”, which are *M* and *SD* (and skew-shape) parameters that can be specified by the researcher elsewhere on the Sources sheet; the distributions are also graphically illustrated in the spreadsheet. Follower count is constrained to be ≥ 0, and credibility scores are constrained to be between 0 and 100.

#### Posts

The Posts sheet allows researchers to customize the stimuli used, including how interactions with each post influence the participants’ follower count and credibility score. As with sources, each post has a unique ID that is not displayed to participants. Each post can have a text headline, content (which can be text or image), or both (see Fig. 1). A static image or animated gif can be included via Insert → Image → Insert an image in the cell. The social media simulator does not currently support the inclusion of longer videos or sound.

Researchers are required to classify each post as true or false in the “Is True” column. However, it is important to note that researchers are not obliged to include both true and false posts, and the “Is True” classification can be used instead for defining other binary categories of posts (e.g., offensive vs. inoffensive; left-leaning vs. right-leaning). If researchers are using the “Is True” classification to define other categories, any reference to true and false information in this paper, and in the Google Sheets template, will instead apply to their chosen classification.

For each post, researchers can specify engagement metrics (i.e., the number of likes, dislikes, shares, and flags the post has ostensibly accrued). Values can also be entered to specify the change to participant follower count/credibility score that will result from different actions (e.g., +3 followers when the post is shared). These parameters can be individually set for a post, or randomly sampled from distributions defined by the “default post values”, which can be seen and edited elsewhere in the Posts sheet and may be set separately for the two levels of the “Is True” classification. Values sampled from the “default post values” distribution will not necessarily be whole numbers; however, information shown to participants will be rounded to the nearest whole number. If multiple responses are enabled, changes in follower count and credibility score will be the aggregated value of all reaction options selected by the participant.

Finally, if comments are enabled in a study, these are also set on the Posts sheet. For each post researchers can include up to three comments. For each comment researchers can set the source of the comment, the content of the comment (i.e., the message), as well as the number of likes and dislikes the comment has ostensibly attracted (see Fig. 1).

#### Source and post selection

The Source & Post Selection sheet allows researchers to control the pairings of sources and posts used in the study. There are four pairing options available in the basic study configuration (i.e., not requiring any additional coding from the researcher): (1) Overall-Ratio, (2) Source-Ratios, (3) Credibility, and (4) Pre-Defined.

The Overall-Ratio selection method allows researchers to set the percentage of true posts to display to participants (with the remainder being false). This percentage is used to probabilistically sample posts. Therefore, this value reflects the average percentage of true posts displayed across the entire sample (the exact percentage of true posts displayed to each participant may differ from this value).^7^ Sources will be selected randomly in this setting and, as such, a source’s credibility may not accurately reflect the content attributed to it. Therefore, this setting may be most appropriate when source credibility is not a variable of interest, or is disabled, in the study.

The Source-Ratios selection method allows researchers to define the probabilistic likelihood of each source used in the study presenting a true post. Sources will again be sampled randomly, and assigned posts will be sampled to meet the true:false ratio specified for each source via its true-post percentage in the Sources sheet (see Fig. 2). For example, if a source’s true-post percentage is set to 100%, the source will always post true posts (as long as there are sufficient true posts available in the Posts sheet); if the true-post percentage is set to 80%, there will be a one-in-five chance that the sampled post is false (again, assuming sufficient true and false posts are available).

**Fig. 3:**
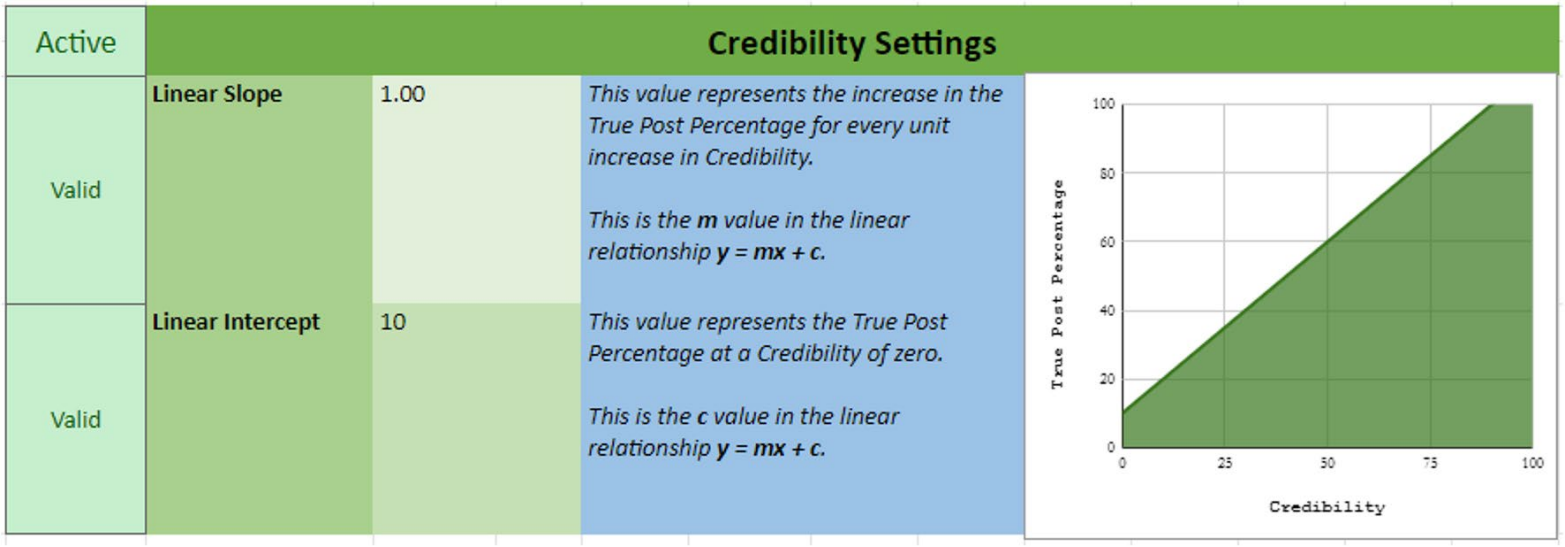
Example of credibility settings in the credibility selection method. Note that, in this example, the relationship between credibility and true-post percentage is set to have an intercept of 10 (i.e., a source with credibility of 0 will be paired with a true post 10% of the time) and a slope of 1 (i.e., with every unit increase in credibility, there is a 1% increase in the likelihood of a true post being sampled). Under these parameters, the likelihood of sampling a true post will reach 100% when credibility is at 90, and as such only true posts will be sampled if a source has a credibility of 90 or above

The Credibility selection method samples posts based on the specified credibility of the sampled source. That is, sources are randomly sampled and posts are then sampled based on the credibility of the respective source. The sampling parameters can be set under the “credibility settings” in the Source & Post Selection sheet (see Fig. 3). When using this option, researchers must set a slope (representing the increase in likelihood of a true post being presented with every unit increase in credibility) and an intercept (representing the likelihood of a source displaying a true post when source credibility is zero). The relationship between credibility and true-post percentage is constrained to be linear (though it can be positive or negative) and both scores are bound by limits of 0 and 100.

Finally, the Pre-Defined selection method allows researchers to fully specify the source-post pairings shown to participants, using the additional Pre-Defined Source/Post Pairs sheet. If this option is selected, researchers must define all source-post pairings for the defined length of the experiment. Researchers can also specify whether they want the posts to be presented in a randomized order; if ‘No’ is selected, posts will be displayed in the exact order specified in the Pre-Defined Source/Post Pairs sheet.

### Deploying studies

Once researchers have configured their study, the Google Sheets file must be downloaded as a Microsoft Excel file. This file can then be uploaded to the administration page of the researcher’s unique study website. Researchers can then enable the study to make it accessible online. We note that the Firebase hosting website has a limited free tier. Should this limit be surpassed, researchers will be charged for any additional usage if they select the Blaze plan in Firebase (this will vary depending on the study parameters, see here https://themisinformationgame.github.io/FirebasePricing for a cost breakdown). If researchers chose to use a Spark (No-cost) Firebase plan studies will stop working appropriately if the daily usage limit is exceeded. Studies including only text will allow for a relatively high number of participants (i.e., several hundred) per day under the free tier; however, the inclusion of images or gifs (especially large, uncompressed images) can reduce this number significantly. We note that increasing the sample size allowance under the Blaze (pay-as-you-go) Firebase plan is relatively inexpensive, however, researchers may wish to consider collecting data over multiple days to avoid daily usage limits under the free tier.^8^

**Fig. 4:**
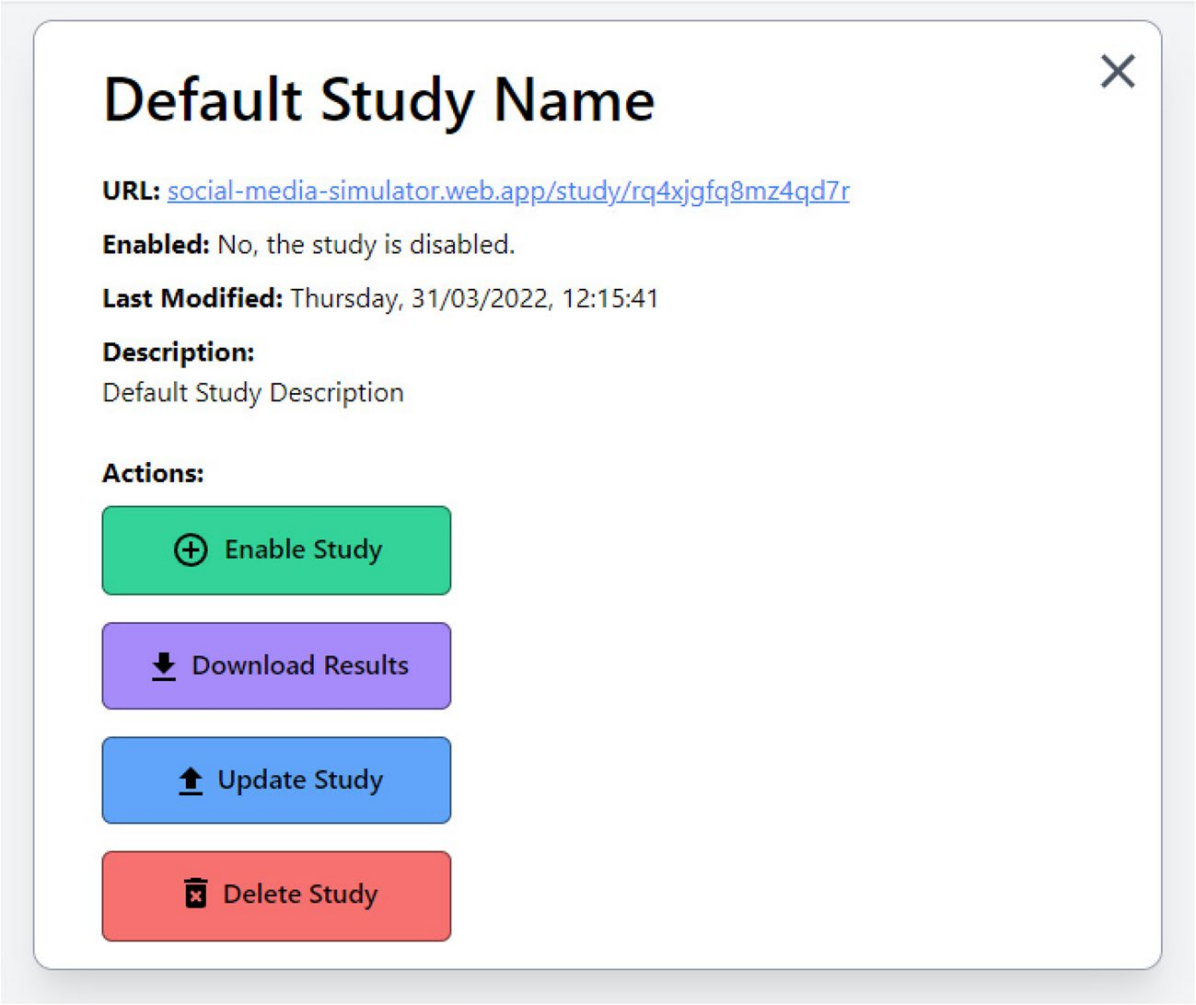
Example administrator study view

To direct participants to a study, the unique study URL can be distributed to participants and accessed on any computer connected to the Internet. Once participants have completed the study, their responses are securely recorded and can be downloaded from the admin page (see Fig. 4).

If researchers wish to collect additional data about participants (e.g., demographic information, belief ratings), allocate participants to different conditions (which can be done by creating two or more variations of the study using multiple Google Sheets templates and uploading each of them to the administration backend of the researcher’s unique website), or allocate participants a unique ID, this can be achieved by linking the study to an online survey. Below we outline four ways to link the Misinformation Game to a Qualtrics survey (Qualtrics, Provo, UT). Each option has certain benefits and limitations.

#### Linking at the end of a Qualtrics survey

If researchers wish to use a Qualtrics survey to administer questions only prior to the social media simulator, the simplest solution is to use the Qualtrics ‘End of Survey’ function to direct participants from the survey to the simulator. Note that when using this method, researchers cannot redirect participants back to the same survey after the component of the simulator. That said, participants can be directed to a separate survey after the social media simulator (although this will lead to the creation of three separate data files per participant: one from the simulator and two from the Qualtrics surveys).

If all participants complete the same version of the social media simulator (i.e., the same condition) this process simply requires researchers to set up an ‘End of Survey’ link (under Survey Flow → Add New Element Here → End of Survey). Under the ‘Customize’ function, select ‘Override Survey Options’ and ‘Redirect to a URL’. Here researchers must enter the Misinformation Game study URL shown at the admin backend of the study (see Fig. 4). The access ID on the first page of the simulation can be automatically populated by adding the URL parameter ?id=<Participant-ID> to the end of the Misinformation Game study URL. If the participant ID is assigned using embedded data in the Qualtrics survey, <Participant-ID> should be replaced with the embedded data set for the participant ID on Qualtrics (e.g., ${e://Field/Participant%20ID}).^9^ Participants can be randomly allocated to different versions of the social media simulator (i.e., different conditions) using the ‘Randomizer’ function of Qualtrics. Specifically, under ‘Survey Flow’, a ‘Randomizer’ element can be added with multiple ‘End of Survey’ elements. Equal sample sizes can be achieved by selecting the randomizer’s ‘Evenly Present Elements’ option.

#### Linking within a Qualtrics survey

The following linking options allow researchers to link to the social media simulator within a Qualtrics survey without ending the survey. The first option requires participants to open the social media simulation in a new browser tab; the other integrates the simulation with the survey such that they appear as a single continuous study. To link multiple study versions (i.e., randomly assign participants to different study conditions), it is recommended that the ‘Randomizer’ function is used as described in the above section; however, instead of randomly selecting an ‘End of Survey’ element, a random block is inserted, with each social media simulator version contained in a unique block.

##### New tab

This option creates a link to the simulator that will open in a new browser tab. This can be done by creating a text/graphic question in Qualtrics, selecting ‘Rich Content Editor’, and then entering the below into the HTML source view^10^:


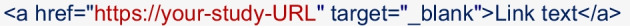


As this option keeps the Qualtrics survey open whilst opening a new tab for the social media simulator, we recommend that researchers create a timing question in the survey that delays the submit button by the minimum time required to complete the social media simulation (to reduce the risk of participants continuing the survey before completing the simulation in the other tab). Additionally, we recommend that the survey should require participants to enter a password or unique completion code given on the debrief page of the simulation, to confirm that they have completed it. We note that using a single hard-coded password can be beneficial, as researchers can use content validation in Qualtrics to stop participants continuing the survey until they have entered the correct password; however, there is a risk that passwords could be shared between participants. By contrast, completion codes are generated to be unique for each participant, and thus cannot be shared; however, their uniqueness also means that content validation cannot be used to stop participants continuing the Qualtrics survey if they enter a false code before (or instead of) completing the social media simulation. However, completion codes can be used to exclude participants retrospectively.

##### Inline frame element

The inline frame (i.e., *iframe*) option allows researchers to embed the social media simulation in the survey. To do so, researchers should create a text/graphic question where they want the simulation to be displayed, selecting which will bring up editing options in the sidebar; from these options the researcher should select JavaScript (under Edit Question → Question Behavior), and enter the following:


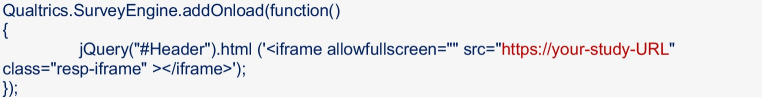


Additionally, the wrapper (container) and iframe classes must be defined in Qualtrics using CSS (via Look and Feel → Style → Custom CSS) and entering the below. Note, the padding can be adjusted to fit the needs of individual projects:


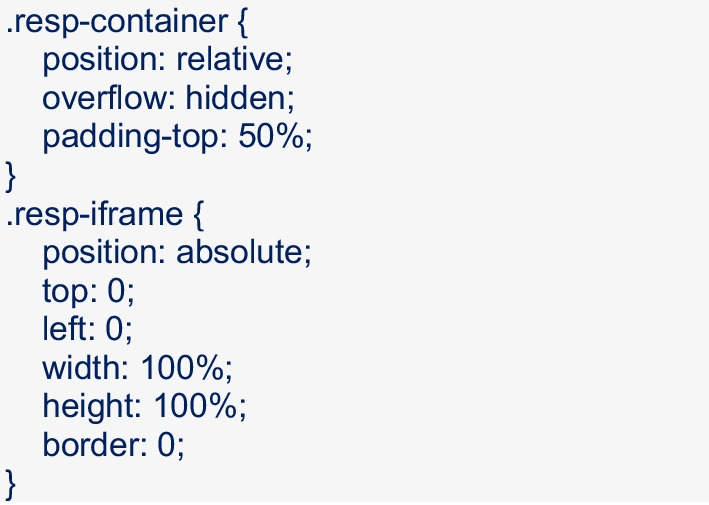


To change the width of the Qualtrics survey (i.e., reduce the white border around the iframe), the following can be entered above the custom CSS specified above (the width percentage can be edited to meet the needs of the specific project):


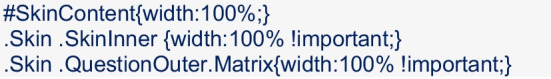


Using this method will mean the social media simulation will be displayed within the Qualtrics survey; however, the iframe will take up the whole screen. It is important to note, the Qualtrics survey and the Misinformation Game cannot “talk” to each other, and thus the Qualtrics survey will not “know” when a participant has completed the social media simulation (rather the participant must self-advance). We therefore recommend researchers add a password or completion code to the survey in line with the methods outlined in the “New Tab” section above. Researchers can require participants to enter the completion code on the page displaying the Misinformation Game by making the question that contains the iframe a text-entry question (we recommend adding text that will prompt participants to add their completion code, e.g., “Enter Completion Code Here:”), and entering the following in the JavaScript field:


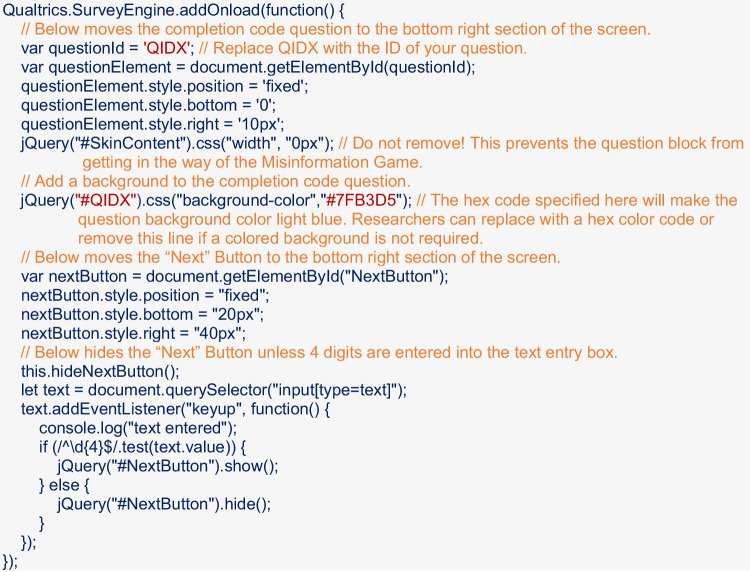


The above code will place the text-entry question for the completion code in the bottom right corner of the screen, and will only display the “Next Button” if four digits have been entered into the text box. This requirement can be removed or edited by researchers to fit their needs. An example Qualtrics study using this integration method is available here https://uwa.qualtrics.com/jfe/form/SV_bqPc1UcnE2uNH8i, and a Qualtrics template (.qsf file) for this integration method is available at https://osf.io/w58ak/.^11^

### Data storage

Results for each study run using the Misinformation Game are securely stored and can be accessed through the Misinformation Game Administrator Dashboard. Results can be downloaded as a Microsoft Excel file using the “Download Results” function (see Fig. 4). Results can only be accessed by the administrator who uploaded the study (i.e., each administrator will have a separate administrator dashboard where only the studies they have created can be accessed). The results spreadsheet contains all information about the posts (including order of presentation and source-post pairings) as well as participants’ reactions (how they engaged, including response times, and any comment information) and the corresponding follower count/credibility score changes. Timing information is also recorded, including (1) time to first interaction (i.e., time between the post becoming visible and the first selection of a response, in milliseconds), (2) time to last interaction (i.e., time between the post becoming visible and the final selection of a response, in milliseconds), and (3) dwell time (i.e., duration of time that the post is visible, in milliseconds). This information is similar to timing information provided by online survey platforms, and thus we recommend using some caution when interpreting the timing data (for discussion of limitations of collecting timing information in online experiments, see Anwyl-Irvine et al., 2021; Barnhoorn et al., 2014). Metadata and general study information, including overall task duration for each participant, are also included in the output. See supplementary materials for an example results output.

### Constraints and related work

Below we outline some constraints of the Misinformation Game, and point to alternative testing platforms that researchers may find more appropriate for their specific research needs.

For one, although the social media simulator is similar to the format of Facebook and Twitter (and to some extent Instagram), it bears little resemblance to other popular platforms (e.g., TikTok, YouTube, WhatsApp). As such, the findings of studies run using the Misinformation Game may not generalize to said platforms. While the design choices were intentional, we note that the worrying proliferation of misinformation on video-sharing social media platforms (e.g., Yeung et al., 2022) warrants the development of other, ecologically valid testing platforms.

Further, the Misinformation Game cannot facilitate interactions between participants, and as such all social information must be simulated by researchers. The social media simulator also does not allow participants to personalize their environment: There is no capacity for participants to create their own page or avatar, or make their own posts (though they can comment on other posts). While this allows for controlled experimental designs, it may limit the immersive and naturalistic nature of the studies run on the platform.

Similar testing platforms have been developed that may better address particular research questions. Most notably, Jagayat and colleagues (2021) developed the Mock Social Media Website tool, a highly realistic tool that closely mimics the features of Facebook and Twitter. This tool also accommodates researchers with relatively limited coding experience and allows for the collection of behavioral data based on how participants interact with posts. It allows for some personalization and facilitates the inclusion of videos. However, this tool does not have several features available in the Misinformation Game (e.g., disabling and enabling of certain reaction options, capacity to provide behavioral feedback, the ability to force responses to posts) and does not provide researchers with the same level of control available from the Misinformation Game. For researchers interested in user-to-user interactions, Community Connect (Mahajan et al., 2021) enables dynamic interactions between participants in a social-media-style format. Although this tool facilitates the investigation of between-user interactions and the social transfer of information, it requires participants (or confederates) to create and engage with the content, thereby reducing experimental control. Additionally, Community Connect is less accessible for researchers with a lower level of coding ability.

## Empirical validation study

As a demonstration of some of the capabilities of the social media simulator and to validate the Misinformation Game as a research tool, we ran a simple study that examines how different behavioral prompts and incentive structures influence engagement behavior and misinformation belief.

Demographic information was collected, and participants were allocated to different experimental conditions, within a Qualtrics survey. Participants were assigned to one of two conditions: In the experimental condition they received a prompt to maximize their follower count (*“Your objective is to maximize your number of followers!”*) and in the control condition they were prompted to behave how they normally would on social media (*“Please interact with posts how you would on social media.”*). The study template was set such that comments and quantity of likes, dislikes, shares, and flags were not displayed, and each post was displayed on a separate page. Participants could gain followers by engaging with posts; this was set such that sharing generally led to a greater increase in followers than the other engagement options, though liking and disliking also attracted followers. Thus, sharing everything was the optimal strategy for participants to maximize their follower count (see Materials below for details). It was predicted that participants in the maximize-followers condition would like and share significantly more posts, including false posts, than those in the control condition, resulting in a significantly greater number of followers at the end of the simulation. At the end of the study, participants were redirected back to the Qualtrics survey where they rated their belief in the claims presented in the simulation. This was done to assess whether any differences in behavior that may arise due to the different behavioral prompts subsequently influenced misinformation belief, potentially due to factors such as need for consistency (Van Bavel et al., 2021).

### Method

#### Participants

Adult participants residing in the U.S. were recruited using the crowd-sourcing platform Prolific. To detect a small effect of *f* = .20, an a priori power analysis with α = .05 and 1 – β = .80 (using G*Power3; Faul et al., 2007) suggested a sample size of 200. To ensure adequate power after exclusions, a total of 210 participants were recruited. Based on a priori criteria, participants were excluded from analysis if they: (1) self-reported their English proficiency as only “fair” or “poor” (vs. “good”, “very good”, or “excellent”; *n* = 0); (2) self-reported lack of effort (*n* = 0); or (3) had a completion time < 4 min (*n* = 1). An additional eight participants were excluded for repeating the simulation multiple times (due to participants refreshing the page after completing the simulation).^12^ The final sample size was thus *N* = 201 (*n* = 102 in the maximize-followers condition; *n *= 99 in the control condition). The sample consisted of 127 females, 71 males, two non-binary individuals, and one with non-disclosed gender; age range was 18–85 years (*M*_age_ = 36.51, *SD*_age_ = 13.90).

#### Materials

**Fig. 5:**
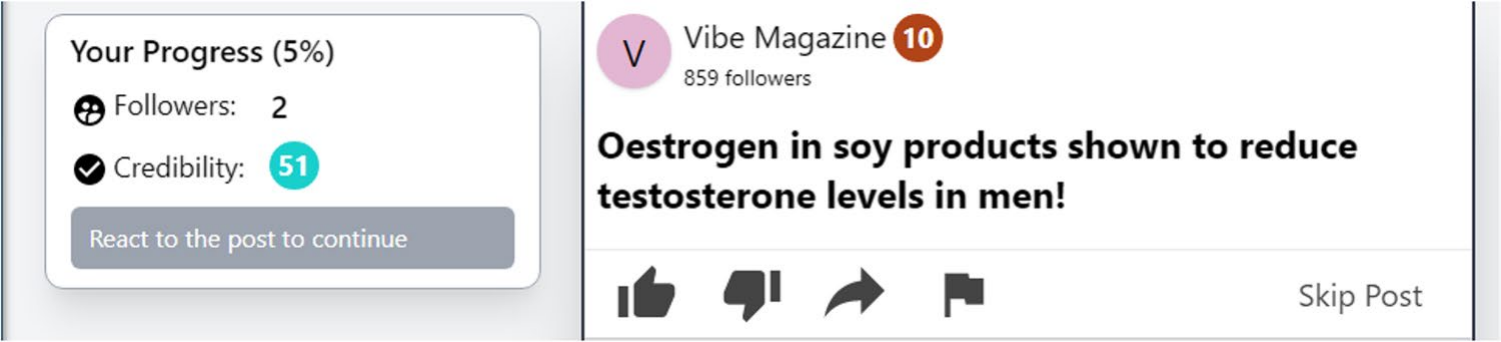
Example false claim used in the validation study

Twenty claims (ten true, ten false) were used as posts in this study. Each claim related to a health issue and was selected to be slightly surprising or counterintuitive (an example false claim was “*Eating chocolate for breakfast can help you lose weight*”). Non-political claims were used to minimize the chance of belief or sharing behavior being influenced by participants’ political orientation or underlying worldviews. Each claim was presented in the social media simulator and attributed to one of ten generically named sources (see Fig. 5 for an example false claim as it appeared in the study). Sources had default “letter-on-circle” avatars. Source follower count was randomly generated from a normal distribution (truncated at 0) with *M* = 500 and *SD* = 500, and source credibility was generated from a normal distribution (truncated at 0 and 100) with *M* = 50 and *SD* = 30. As such, source follower count and credibility score varied between participants. The source-and-post-selection method was set to “Credibility” with intercept of 0 and slope of 1, meaning that source-post pairings also varied between participants, but in general true posts were more likely to be paired with more credible sources. Presentation of sources was constrained such that each source appeared a maximum of four times per participant (given this, the subset of sources seen by each participant varied slightly). See supplementary materials for a full list of true and false claims, as well as more detailed source information.

**Fig. 6:**
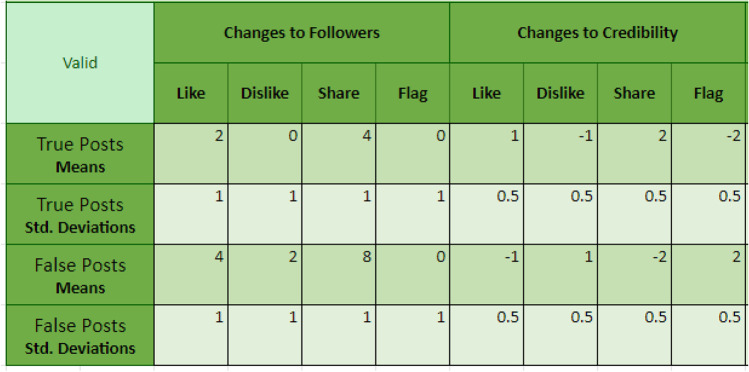
Distribution parameters for changes to follower count and credibility in the validation study. Under these parameters the maximum possible followers a participant could obtain in the study was approximately 120

Participants could interact with each post either by endorsing (i.e., liking or sharing it) or dis-endorsing (i.e., disliking or flagging). Participants could also choose to “skip post” if they did not wish to interact with the post. Changes to participants’ follower counts and credibility scores were randomly sampled from distributions set using the default post values shown in Fig. 6. These values were chosen so that positively engaging with both true and false posts would increase follower numbers, with a stronger effect for false posts than true, and a stronger effect for sharing than liking. Dis-endorsing posts on average did not change follower numbers, apart from disliking false posts, which on average increased followers slightly.^13^ This created an information environment where participants who positively engaged with (and in particular: shared) content attracted followers, especially if the information was false. The participants’ credibility scores increased with liking and sharing true posts, and decreased with liking and sharing false posts; the opposite pattern was implemented for negative interactions (disliking, flagging). Skipping posts resulted in no change to a participant’s follower count or credibility score.

Belief in the claims was measured in a Qualtrics survey using direct ratings (*“Please indicate how much you believe the following claim to be true or false”*) on an 11-point Likert scale ranging from 0 (“certainly false”) to 10 (“certainly true”).

#### Procedure

**Fig. 7:**
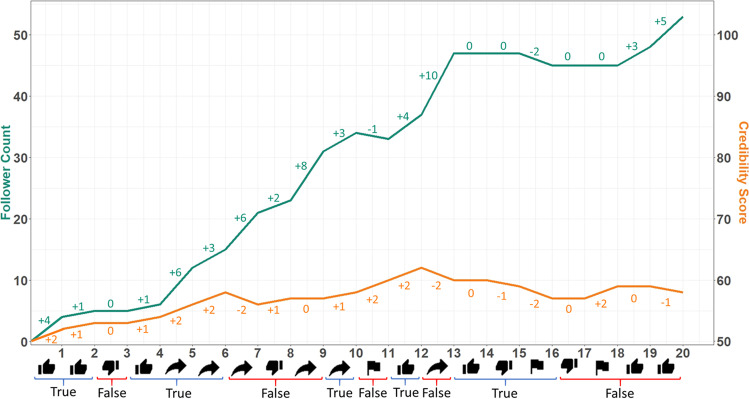
Example participant data over task trials. The participant with the ID 52194 was used in the above example. The *green line* depicts change in follower count, and the *orange line* depicts change in credibility over trials. Initial credibility score was 50, and as such the credibility-score *y*-axis (*right*) was truncated to reflect this. Over the experiment, participant follower count rose from 0 to 53, and participant credibility score rose from 50 to 58. Post type (i.e., true or false) and participant reaction choice are displayed at the base of graph. Note that participants were restricted to a single reaction to each post.

Participants received an ethics-approved information sheet and provided their age, gender, and English proficiency in a Qualtrics survey before being randomly assigned and redirected to one of the two conditions in the social media simulator. Prior to beginning the simulation, participants received instructions on how to interact with posts, as well as a behavioral prompt instructing them to either maximize their follower count or behave normally as they would on social media, depending on which condition they were assigned to. Participants were then exposed to all claims on separate pages and in a randomized order with randomized sources, and were required to interact with each post (i.e., like, dislike, share, flag, or “skip post”). Their follower count and credibility scores were then updated depending on their choice before the next post was displayed. See Fig. 7 for an individual participant’s engagement behavior and change to follower count and credibility score across the course of the simulation.

Upon completing the social media simulation, participants were redirected back to the Qualtrics survey, where they were asked to rate their belief in each claim presented in the simulation. Participants were then asked whether their data should be excluded due to lack of effort, after which they were fully debriefed about the purpose of the study, and explicitly told which claims were false. Median completion time was 7.1 min and participants were compensated £0.88 (approx. US$1.10 at the time of the study) for their time.

### Results

**Fig. 8:**
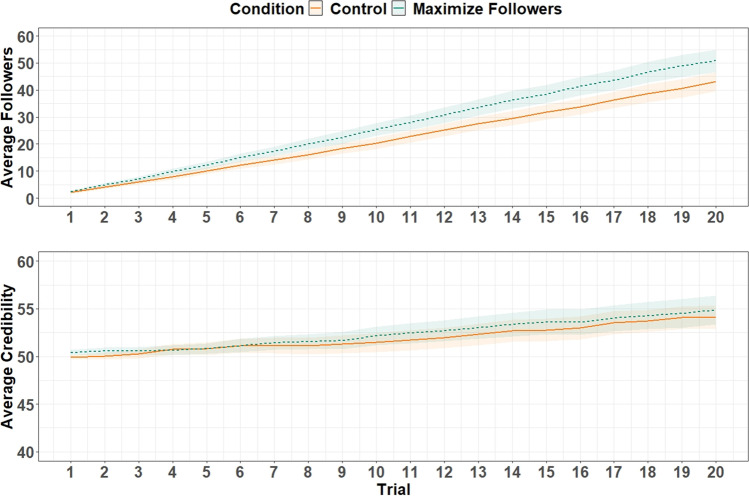
Mean follower count (*top*) and credibility score (*bottom*) over trials across*. *Participant credibility scores began at 50, whereas follower count began at 0. Due to this, the credibility *y*-axis was truncated at 40. *Shaded areas* represent 95% confidence intervals

To determine whether participants in the maximize-followers condition followed the behavioral prompt, we first ran an independent samples *t* test contrasting follower count in the final trial across the two conditions. Final follower count in the maximize-followers condition, *M* = 50.78, was significantly greater than in the control condition, *M* = 43.10, *t*(199) = 2.79, *p* = .006, *d* = .39, suggesting that participants followed the behavioral prompt. We do note that single-sample *t* tests showed that follower count increased substantially over the task in both conditions, with large effects observed in the maximize-followers condition, *t*(101) = 24.52, *p* < .001, *d* = 2.43, and the control condition, *t*(98) = 23.94, *p* < .001, *d* = 2.41. We additionally ran an independent-samples *t* test to assess whether there was any difference in final credibility scores across the two conditions. There was no statistical evidence of a difference in the final credibility scores between the maximize-followers condition, *M* = 54.88, and control condition, *M* = 54.13, *t*(199) = .76, *p* = .447, *d* = .11, though single-sample *t* tests revealed that the credibility scores significantly increased over the task in both the maximize-followers condition, *t*(101) = 6.34, *p* < .001, *d* = .63, and the control condition, *t*(98) = 6.79, *p* < .001, *d* = .68. Mean changes in follower count and credibility over time are illustrated in Fig. 8.

To test what drove the difference in follower count across the two conditions, Kruskal–Wallis tests were run contrasting frequency of each engagement type across conditions. The mean number of each reaction type across the two conditions and the true and false posts is presented in Table 1. Note that the maximum possible number of reactions for a single post type (i.e., true, false) in each condition is 10. Although participants in both conditions shared more true posts than false posts, participants in the maximize-followers condition shared significantly more true, *H*(1) = 8.89, *p* = .003, ε^2^ = .04, and false, *H*(1) = 5.29, *p* = .021, ε^2^ = .03, posts than participants in the control condition. There was no significant difference in the quantity of other engagement types for either true or false posts across conditions, suggesting the increased follower count in the maximize-followers condition was primarily driven by people choosing to share more posts.

**Table 1:** Mean number of reactions across conditions and post types

Condition	Post type	Reaction type				
		Like	Dislike	Share	Flag	Skip
Control	True	4.32	1.98	1.55	0.76	1.39
	False	3.17	2.81	1.22	1.48	1.31
Maximize followers	True	3.85	1.83	2.62	0.65	1.05
	False	2.83	2.45	2.06	1.71	0.95

Finally, to assess whether there was any difference in claim belief across the two conditions, we ran independent samples *t* tests on the belief data. There was no significant difference in true claim belief between the two conditions (*M* = 5.80, *SD* = 1.45 in the maximize-followers condition; *M *= 5.65, *SD* = 1.34 in the control condition), *t*(199) = .76, *p *= .450, *d* = .11. There was also no difference in false claim belief between the two conditions (*M* = 4.29, *SD* = 1.29 in the maximize-followers condition; *M *= 4.21, *SD* = 1.47 in the control condition), *t*(199) = .38, *p *= .704, *d* = .05. A paired-samples *t* test shows that participants in both conditions believed true claims more than false claims, *t*(200) = 11.68, *p *< .001, *d* = 1.06 (conditions collapsed).

### Discussion

Our results demonstrate that the study instructions led to a change in participants’ behavior: specifically, those instructed to maximize their number of followers chose to share significantly more true and false posts than those in the control condition, consequently attracting more followers. We note that across both conditions the average number of posts shared was relatively low (on average, less than 20%). However, the small number of trials may have limited the extent to which participants could learn that sharing would attract more followers than the other options, and then increase their rate of sharing before the social media simulation ended. The fact that sharing was lower in the control condition, where participants were instructed to behave as they normally would on social media, also indicates that “normal” behavior on social media involves a low degree of sharing, and suggests that people may not normally be so motivated to attract followers (or do not expect to attract followers by sharing others’ posts).

There was no evidence of difference in claim belief between the two conditions, for either true or false claims. This was despite the higher rate of sharing in the maximize-followers condition, indicating that participants’ beliefs and behaviors did not fully align, thus supporting the notion that under certain conditions people may propagate misinformation they do not believe (see also Ren et al., 2023). However, participants believed and numerically shared true claims more than false claims, indicating some alignment. Participants were suspicious in general, with the average belief rating around the mid-point on the scale even for true claims; this may have contributed to the low rate of sharing.

Participants distinguished between true and false claims, believing true claims more than false claims in both conditions. More frequent sharing and liking of true claims than false claims meant that participants’ credibility scores tended to increase over the task (whereas equal sharing/liking of true and false claims would keep credibility around 50). The fact that credibility scores did not differ between the conditions indicates that the preference to share and like true over false claims was similar, despite the fact that participants in the maximize-followers condition generally behaved to maximize followers and sharing false claims was actually more effective for attracting followers than sharing true claims. It may be that participants were sensitive to their credibility scores (or external norms not to share false information) and were able to find a compromise between following the instructions (i.e., maximizing their follower count) and maintaining an acceptable level of credibility.

Broadly, the results returned by the social media simulator study are sensible and consistent with past research (e.g., Ren et al., 2023). Participants engaged with the simulator in a meaningful way and were sensitive to the dynamic feedback provided across trials, updating their engagement behavior in line with the prompts. These results suggest the simulator is a valid tool for research in the misinformation realm. This said, our results do not speak to participants perception of the social media simulator, such as whether they found it to be realistic. To gain some insight into how the simulator is perceived by participants, we ran a user-experience study, which is described next.

## User-experience study

**Fig. 9:**
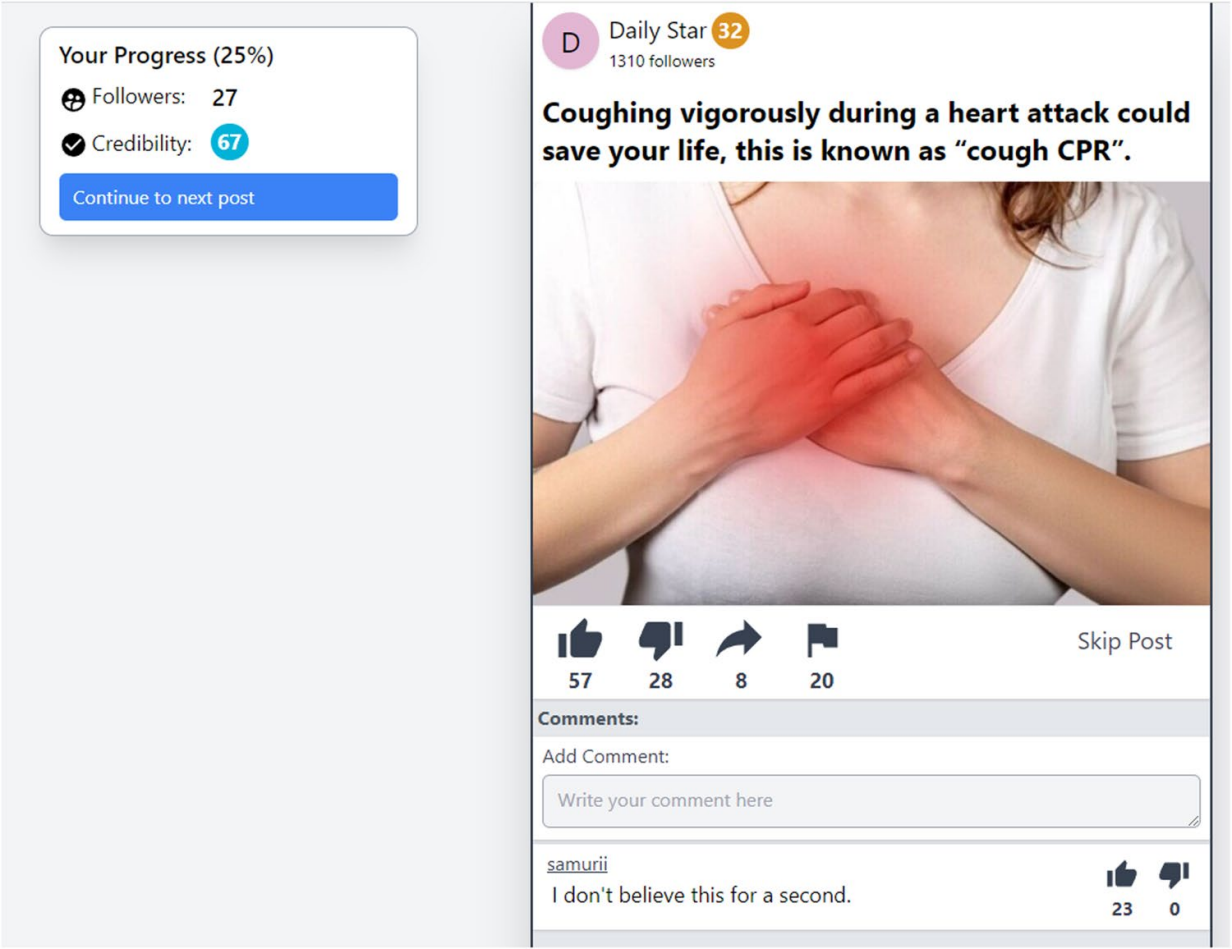
Example false post presented in the user-experience study. Image used taken from: *Heart attack concept, woman suffering from chest pain* [Photograph], by M. Verch, 2021, Flickr, https://www.flickr.com/photos/30478819@N08/51096039583 CC BY

To assess participants’ perceptions of the platform’s usability and realism, a second study was run with all of the Misinformation Game’s features enabled, and with the inclusion of images alongside posts (see Fig. 9 for an example post). In this study, each post was presented on a separate page (rather than in a feed), and as such the perceived realism reported below may be slightly lower than what would be observed in studies using the feed function.

**Fig. 10:**
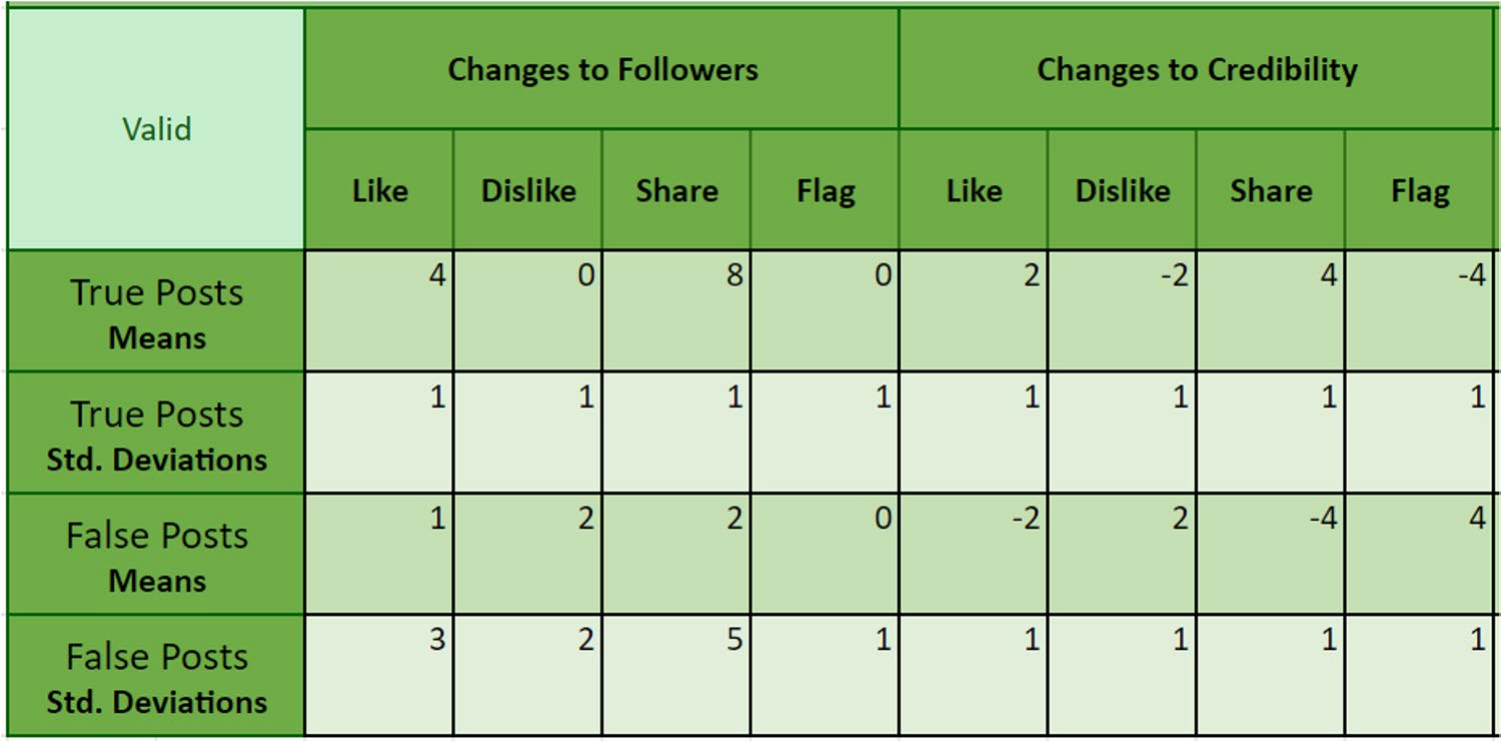
Distribution parameters for changes to follower count and credibility score in the user-experience study

Due to resource limitations, the sample size was small and did not include people from a diverse population of interest (e.g., younger vs. older adults, frequent vs. infrequent Internet users). As such, we note that the following findings simply provide a proof-of-concept, and we caution against overgeneralizing. Specifically, *N* = 48 Prolific workers (29 females, 17 males, two non-binary individuals; *M*_age_ = 33.81, *SD*_age_ = 14.58, age range = 18–83) were exposed to the same 20 claims and sources as in the empirical validation study (but with images and comments added). Sources and posts were again paired using the “Credibility” method, and the parameters used for follower count and credibility changes are shown in Fig. 10. During the social media simulator component of the study participants were prompted to behave how they normally would on social media. Engagement with each post was delayed by 1.5 s to reduce inattentive responding. If participants did not wish to interact (i.e., engage) with a post they could simply continue to the next post after a 1.5-s delay without engaging (including skipping).

After completing the social media simulation, participants responded to a six-item questionnaire that assessed the social media simulator’s ease of use (“*the simulation felt easy/confusing”*), engagingness (“*the simulation felt engaging/boring”*), and realism (“*the simulation felt realistic/unnatural”*); responses were measured on a five-point Likert scale from 0 (“Strongly Disagree”) to 4 (“Strongly Agree”). Following the questionnaire, participants were given the option to provide written feedback about the simulation, after which they were fully debriefed.

**Fig. 11:**
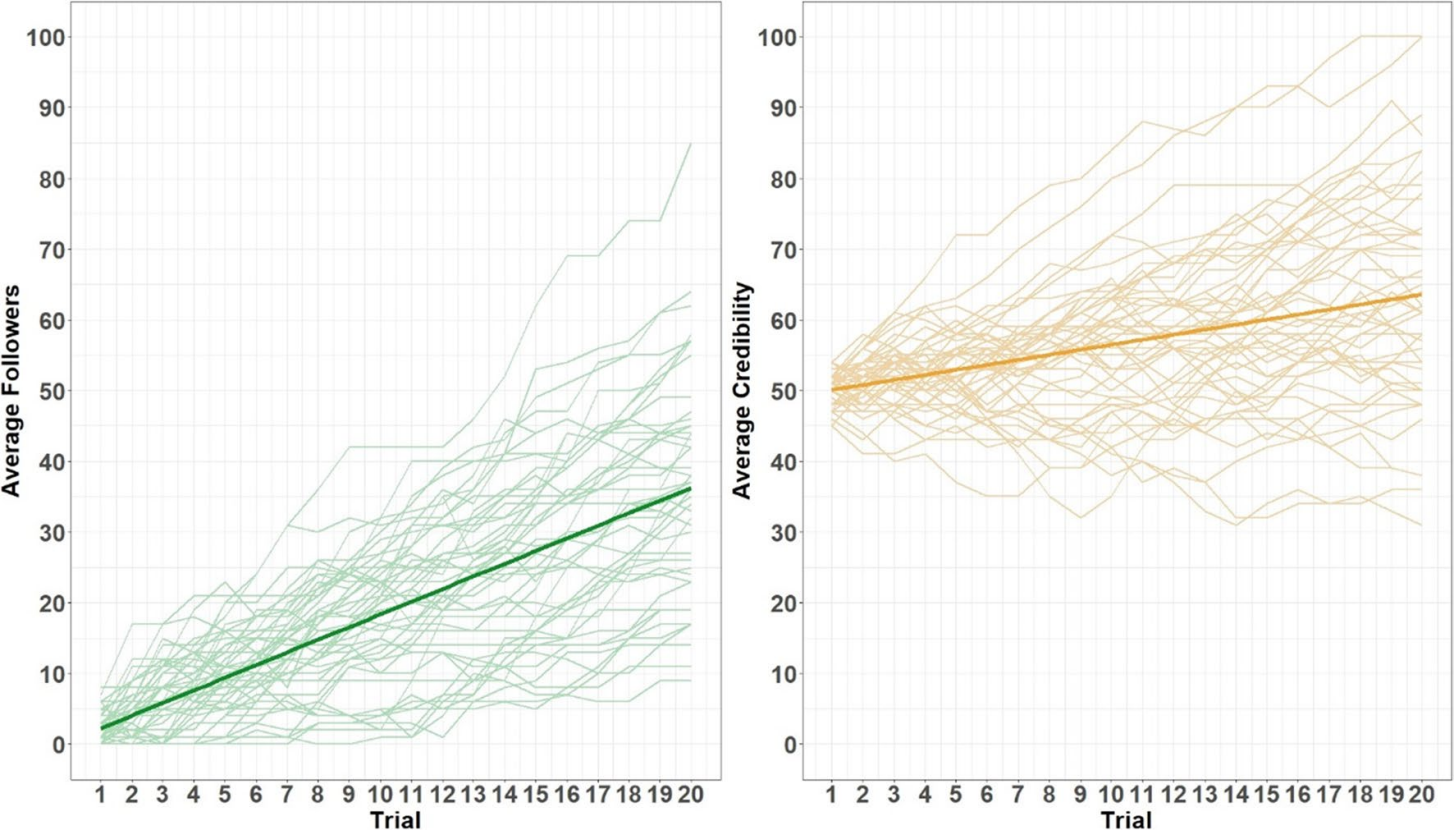
Follower counts and credibility scores across trials, plotted for each participant. Bolded lines represent linear model fits.

To check whether participants engaged with the social media simulator as intended, we first assessed trends in participant-engagement behavior. Changes to follower count and credibility over trials are shown in Fig. 11. All participants’ (simulated) follower count and credibility score changed over the course of the study (increasing on average), suggesting that participants interacted with posts in a reasonable manner throughout the study (i.e., they did not continue without reacting on every post). In fact, on average participants engaged with (i.e., liked, shared, disliked, or flagged) 83.1% of the posts they were shown. Additionally, participants left a total of 142 comments on posts (i.e., *M* = 2.96 comments per participant), and reacted to the simulated comments 111 times (103 likes, eight dislikes; i.e., *M* = 2.31 comment reactions per participant). This response pattern indicates that the average participant interacted meaningfully with the social media simulator, even though there was no specific requirement to do so.

**Fig. 12:**
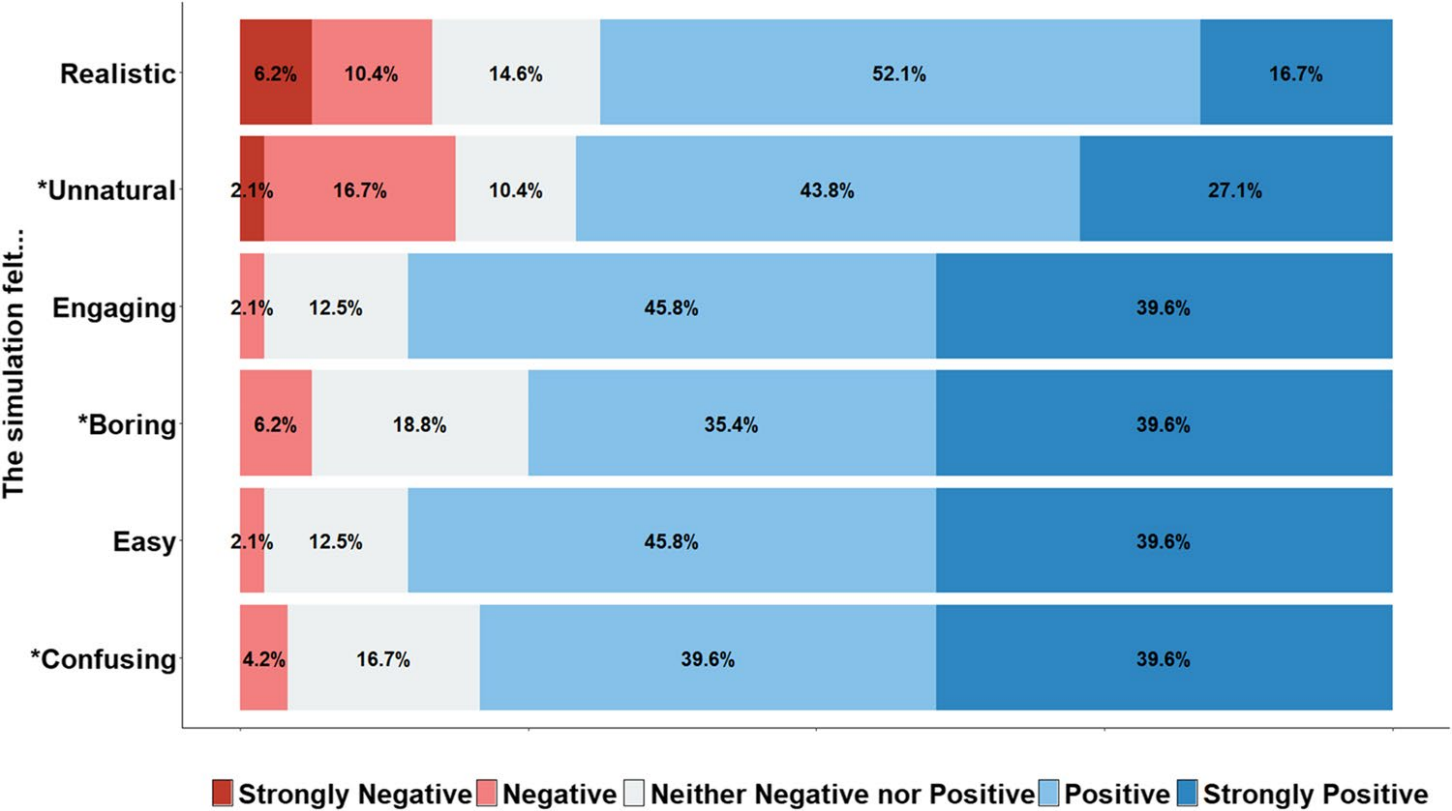
Agreement with the six questionnaire items in the user-experience study. Asterisks denote negative attributes. Note that, to aid interpretation, the scale has been adapted to range from “Strongly Negative” (denoting “Strongly Disagree” for positive attributes, and “Strongly Agree” for negative attributes) to “Strongly Positive” (denoting “Strongly Agree” for positive attributes, and “Strongly Disagree” for negative attributes). No participants responded in a strongly negative manner to the statements “The simulation felt easy/confusing”, or “The simulation felt engaging/boring”

Participant responses to the six questionnaire items relating to user experience are shown in Fig. 12. The first two items relate to realism, the second two items relate to engagingness, and the final two items relate to ease of use. Averaging across the two items per dimension (after reverse-scoring the negatively coded questions), perceptions of the social media simulator were very positive: 82.2% of participants agreed that the simulator was easy to use, and 80.2% agreed that the simulator was engaging. We note that realism was rated slightly lower, with 69.8% of participants agreeing that the social media simulator felt realistic. This was expected, given (1) the general limitations of simulated environments (e.g., the inability to mimic real-life social connections), and (2) the specific constraints of the platform, such as the nature of the follower count/credibility change, as well as the presentation of a single post per page.^14^

Eight participants chose to provide written feedback. The feedback was largely positive, while also providing some insights into the areas where the social media simulator and social media diverge (see Table 2 for all feedback). Specifically, participant P738643 highlighted that people may be more likely to engage with posts in the simulator than on social media, which may limit the ability to draw certain inferences about real-world behavior. This demand effect, however, is unlikely unique to the current paradigm, but rather a general limitation of experimental designs that assess social media behavior. Additionally, we note that the parameters for credibility and follower-count change (P846892), as well as the claims used in this study (P219815), may have appeared unnatural to some—although this is a limitation of the current study and not the platform per se, the feedback emphasizes the importance of keeping realism in mind when developing studies using the Misinformation Game.

**Table 2:** Written feedback provided by participants in the user-experience study

Participant ID	Comment
P219815	*“Some statements were ridiculous”*
P858260	*“No [feedback], fun study”*
P707006	*“Interesting and fun!”*
P738643	*“The simulation layout overall seemed pretty realistic to me, however, it was hard to escape the fact that I was looking at a simulator and not a real platform.* *This made the way I interacted with it not entirely realistic because when using a real social media platform I would just skip over a lot more posts (normally I’d just skip anything that isn’t a friend posting) than I did in the simulator, as I didn’t want to just skip everything.”*
P679933	*“Loved it, don’t know what else to say...* *Thank you and have an amazing rest of your day!”*
P260498	*“I had a lot of fun actually. Wish I could have done more!”*
P846892	*“You wouldn’t lose that fast followers if you post a comment about any subject, regardless of your views. My opinion only, not a fact”*
P464233	*“That was very accurate to the Facebook platform, unfortunately.”*

Although the responses from participants were generally positive, it is somewhat unclear what participants were using as a frame of reference when responding (i.e., whether they were comparing to similar studies using less ecologically valid measures such as a questionnaire, actual social media platforms, or some combination of both). As such, based on what participants were using as a comparison may have led them to over (or under) report how realistic they perceived the platform to be. Irrespective of the comparison used by participants, however, the results provide evidence that the paradigm has improved face validity compared to typical ways of experimentally measuring social media behavior.

## Conclusion

In this paper we presented the Misinformation Game, an open-source social media simulator designed for online behavioral research. As outlined, the social media simulator can be easily customized to fit an array of research needs via the use of a Google Sheets template, and thus is an accessible tool for researchers with all levels of computer literacy. The validation study outlined one potential use of the current platform, specifically to assess how behavioral prompts can influence participants’ engagement choices. Though a simple demonstration, this research paradigm could be expanded using the current platform to help inform the use of prompts that prime information veracity to reduce the spread of misinformation on social media (i.e., accuracy prompting; Pennycook & Rand, 2019). Notably, the results of the validation study suggest that people are sensitive to the dynamic feedback provided by the social media simulator, supporting the use of the tool in assessing how the parameters/characteristics of information ecosystems (i.e., the choice architecture) influence behavior (Avram et al., 2020; Borah & Xiao, 2018; Kozyreva et al., 2020; Lorenz-Spreen et al., 2020). We hope that researchers can leverage the degree of control the social media simulator provides in these areas, a degree that is not possible on social media, to provide insight into the conditions that drive or inhibit misinformation propagation and belief. Ideally, studies using the social media simulator will be able to generate findings that can be used to effect change in these areas, potentially reducing or counteracting the spread of misinformation online (Kaur et al., 2018; Qiu et al., 2017).

Although the Misinformation Game was developed primarily for use in misinformation research, we note again that it has clear utility in other research areas. For instance, the social media simulator could be used to assess how people process other forms of information (or information characteristics) on social media, such as that which is highly emotive (Schreiner et al., 2021), politically valenced, or socially relevant. This would be useful to gain insight into the features of information and information environments that promote engagement more broadly, as well as how to effectively counteract other forms of potentially noxious information, such as offensive or derogatory content.

## Footnotes

1 Here we use misinformation to describe any false or misleading information presented as true.

2 In fact, a correlation of *r* = .44 has been observed between hypothetical sharing of false headlines by MTurk workers in an experimental setting and actual sharing of the same headlines on Twitter (Mosleh et al., 2020).

3 We note that additional misinformation research is of course conducted on real-world platforms (e.g., Grinberg et al., 2019; Margolin et al., 2018; Mosleh et al., 2021; 2022; Pennycook et al., 2021; Shao et al., 2018). However, in addition to potential ethical and legal concerns, this research requires specific data-science skills and comes with issues associated with causal inference and experimental control (e.g., see Aral & Eckles, 2019; E. E. Chen & Wojcik, 2016; K. Chen et al., 2021; Fiesler & Proferes, 2018; Murphy, 2017; Samuel & Buchanan, 2020; Sloan et al., 2020).

4 Any letters and numbers can be entered into the Participant ID box to enter the example games.

5 If researchers wish to use the platform for multiple purposes, or if certain information should not be revealed prior to debriefing (e.g., that the study contains misinformation), we recommend using an ambiguous URL.

6 If posts are displayed in a feed, it is not possible to require participants to interact with all posts.

7 Note that a sufficient number of true and false posts will therefore need to be provided to achieve the defined ratio. If the number of true (false) posts that exist in the design is less than the number of total posts to be displayed, the Overview sheet will produce a warning that, depending on the chosen ratio of true:false posts, a false (true) post may be shown when a true (false) post should be shown (i.e., if the ratio requires a participant to be shown more true posts than exist, a false post will be shown instead, and vice versa).

8 We note that this is relatively inexpensive: One dollar increases the possible sample size by approximately 9.5 times the free tier allowance (e.g., from 650 to 6150 participants).

9 Note this method can be used for all Qualtrics linking options outlined. If directing participants straight from Prolific, in Prolific researchers should select “I’ll use URL parameters”, and add ?id={{%PROLIFIC_PID%}} to the Misinformation Game study URL provided.

10 Note that “your-study-URL” should be replaced with the specific study URL provided; however, it must include the “https://”.

11 An additional option does exist which redirects participants from the Qualtrics survey to the Misinformation Game, and then back to the next page of the Qualtrics survey. However, we have decided to not recommend this option as it caused unanticipated technical issues resulting in data loss for participants who did not have cookies enabled on Qualtrics.

12 This issue was due to an integration error that we have now resolved.

13 We note that the mean change to the follower count for disliking false posts should have been set at *M* = 0; however, this value was incorrectly entered during the design of the study. This error occurred across both conditions.

14 Displaying posts in a feed was added as feature to enhance the realism of studies run using the Misinformation Game. However, this addition was made after the user-experience study had been run.
